# Safety beyond Sight: Handheld Metal Detectors as Diagnostic Allies in the Management of Children Suspected to have Ingested Foreign Bodies

**DOI:** 10.3390/diagnostics14040356

**Published:** 2024-02-06

**Authors:** Tomaz Krencnik, Tadej Jalsovec, Martina Klemenak, Petra Riznik, Jernej Dolinsek

**Affiliations:** 1 Department of Pediatrics, University Medical Centre Maribor, 2000 Maribor, Sloveniajernej_dolinsek@hotmail.com (J.D.); 2 Medical Faculty, University of Maribor, 2000 Maribor, Slovenia

**Keywords:** handheld metal detector, foreign body ingestion, children

## Abstract

Background. Foreign body (FB) ingestion remains a common cause of pediatric emergency department referrals, and the gold standard for detection is whole-digestive-tract radiographic examination. Our study explores whether handheld metal detectors (HHMD) can effectively identify the presence and location of ingested metal objects, potentially reducing the need for additional radiographic examination. Methods. We collected medical data from children with suspected metal FB ingestion who were referred to our emergency department (October 2017–March 2023), focusing on object type and correlating metal detector findings with radiographic images. Results. Data from 43 children (39.5% female; mean age: 4 y) referred to our emergency department were analyzed. Coins (32.6%), button batteries (18.6%), and hairpins (11.6%) were the most common ingested objects. Metal detectors detected the presence of FBs in 81.4% of cases (sensitivity: 89.7%; specificity: 100%). Radiographs, taken for 40 children, showed that the most common locations were the stomach (37%) and intestine (33%). The metal detector signals matched the radiography results in 69.8% of cases. According to HHMD, 34.9% of objects were accessible via endoscopy, contrasting with 51.2% via radiography (*p* < 0.05). Conclusion. While the findings obtained using handheld metal detectors often correlate well with radiograph findings in detecting metal FBs, for an important number of children, this confirmation is lacking, especially when determining the exact location of an object.

## 1. Introduction

Foreign body ingestion is prevalent among children. It was found that 75% of foreign body ingestions in the USA in 2000 corresponded to children 5 years of age or younger [1]. Unlike intentional ingestion among older individuals, most cases for children are accidental. Common household items are often involved [1,2,3]. Up to 85% of reported ingestions involve metal foreign bodies, and coins are the most frequently ingested objects, followed by sharp objects (e.g., pins, screws, and nails), button batteries, and magnets [2,4,5,6]. These objects may become lodged in the esophagus, leading to various symptoms such as drooling, coughing, choking, vomiting, dysphagia, and chest or neck pain [4,7]. The potential complications of retained esophageal coins include perforation, mediastinitis, abscess formation, fistula development, or bleeding. Recognizing symptoms is crucial for prompt medical intervention and the prevention of complications [2,4,8]. Foreign bodies in the stomach tend to pass without complication. However, the rate of obstruction and perforation can rise from less than 1% up to 15% to 35% with the ingestion of multiple magnets or sharp foreign bodies [5,7,9,10,11].

Radiography is the gold standard for the definite diagnosis and localization of ingested foreign bodies, offering insights into size and approximate location, especially for radio-opaque objects (10%) [1,2,4,12]. Yet, radiolucent foreign bodies pose a challenge, as standard imaging methods like radiography and ultrasonography often prove unsatisfactory in pinpointing their locations. The use of opaque contrast agents and magnetic resonance imaging is discouraged. While CT imaging is preferred in certain cases, the associated higher irradiation dose in children necessitates careful consideration [1]. Despite radiography being the preferred method, concerns arise among parents about the additional irradiation exposure. Physicians in rural areas may face constraints in performing radiography due to technical issues. In such scenarios, alternative techniques, like the use of handheld metal detectors, may come into play for determining the presence and location of a metal object. For objects posing higher complication risks, such as button batteries or sharp items, emergent endoscopic intervention is often necessary. Deciding on the need for endoscopic removal or a conservative outpatient approach involves considering factors like the affected child’s age, the time of ingestion, and the object’s size, characteristics, and location [1,9,13].

A handheld metal detector (HHMD) is a portable device that measures changes in the inductance of a coil to determine whether a metallic object is near. The presence of such an object is presented as an audio and/or visual signal. Adjusting the detector’s sensitivity and conducting multiple screenings across various areas enables the potential localization of metal foreign objects within the body [2]. Numerous studies have been undertaken to evaluate the potential of handheld metal detectors in detecting metal foreign bodies, with a specific emphasis on minimizing the need for conducting radiographic examinations on children. These investigations highlight the utility of HHMDs in effectively identifying metallic foreign objects in both children and adults [2,5,14,15,16,17,18].

Endoscopic removal is the method of choice for removing FBs from the upper gastrointestinal tract. Experiences from the pre-endoscopic era show that over 80% of ingested FBs pass through the gastrointestinal tract without causing significant damage or complications [19,20,21]. Even though endoscopic removal is recommended, the optimal timing for the endoscopic removal of ingested FBs in children hinges on several key parameters. Considerations include the affected child’s age and body weight, their clinical presentation, the time elapsed since ingestion, the timing of their last meal, and the type, size, and shape of the FB, as well as its current location in the gastrointestinal tract. Esophageal button batteries require immediate removal, irrespective of symptoms, due to their potential to cause severe complications. Coins, magnets, or sharp FBs lodged in the esophagus necessitate prompt removal, especially within 2 h for symptomatic cases and 24 h for asymptomatic instances. When dealing with single or multiple magnets and metallic FBs beyond the stomach, symptomatic children should undergo consultation with a pediatric surgeon for possible surgery. Asymptomatic children, in the same scenario, may be monitored through serial X-rays to assess progression. Additionally, endoscopic removal is recommended for sharp or pointed, long or large, and wide FBs located in the esophagus or stomach. This comprehensive approach ensures that the decision-making process is informed by a nuanced understanding of each case, prioritizing prompt intervention when necessary to mitigate potential complications [9,19,22].

The aim of our study was to determine whether a handheld metal detector could effectively determine the presence and location of ingested metal foreign objects to a degree that would eliminate the need for radiographic examination. We also tried to find out if there is a difference in the detection rates between different metal FBs.

## 2. Materials and Methods

**Graph 1:**
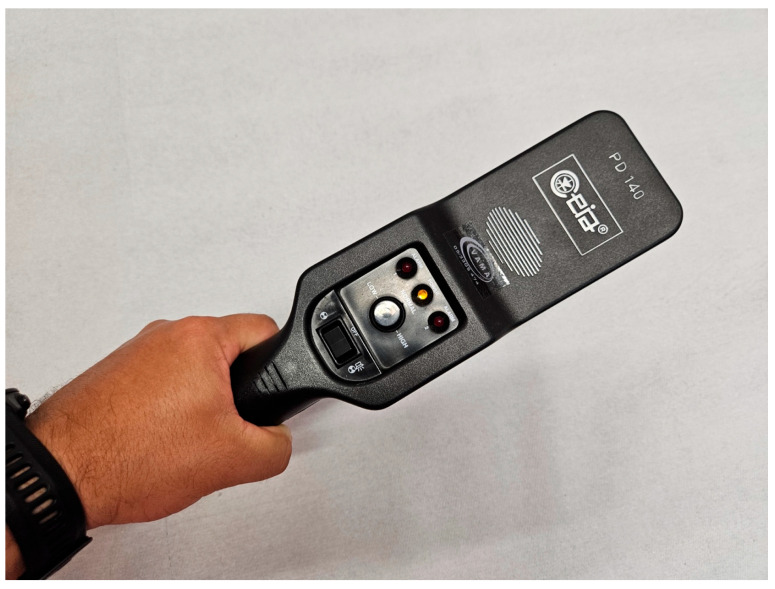
Ceia^®^ PD 140, the handheld metal detector used in our study.

**Graph 2:**
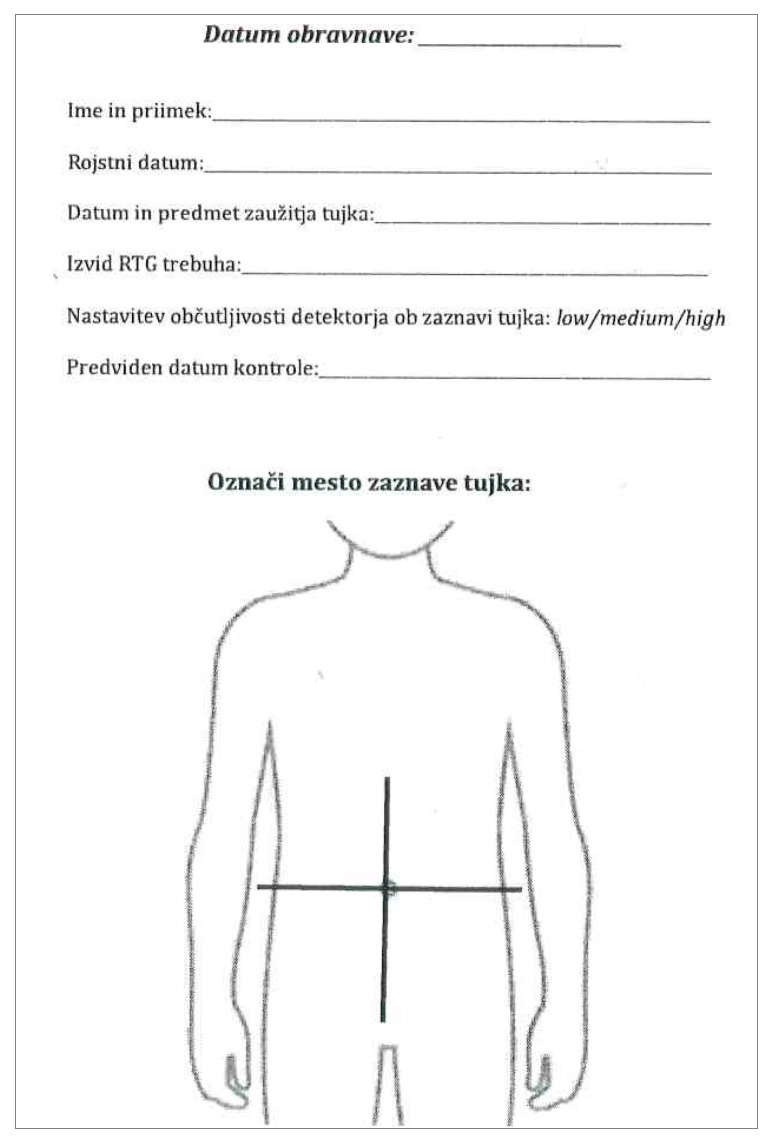
Form for data collection.

We prospectively collected medical data from children less than 19 years old with suspected metal object ingestion who were referred to our emergency department from October 2017 to March 2023. Medical history was obtained, and the presumed time of foreign body ingestion was determined. Following this, a physical examination was conducted in conjunction with the utilization of a metal detector to identify the potential presence and location of a suspected foreign body. The metal detector used, operating at very low frequencies, consists of a receiver and transmitter. Its operation involves detecting changes in an electromagnetic field resulting from the proximity of metal objects. Upon detection of a metal object within this field, the disturbed electromagnetic equilibrium prompts the sensor to activate an audiovisual alarm on the device [2,23]. To standardize the examination process, our physicians utilized the Ceia^®^ PD 140 handheld metal detector, renowned for providing both audio and visual signals upon metal detection (Graph 1). Before the utilization of the HHMD, each child was undressed so that he/she was wearing only undergarments, which were required to be free of any metal objects. Preferably, we advised the parents to fully undress their children. We performed the screening with the HHMD in the middle of the examination room, far away from all metal objects in the room. The examinators were instructed to identify all metal objects on the person holding the child so that these were removed before the start of the screening process. Physicians were then instructed to examine the child by performing several passes with the HHMD, moving from the periphery towards the bellybutton. Upon receiving a signal, they were further guided to refine the location using additional passes and by adjusting the sensitivity of the device. The findings were then marked on the specially prepared forms, where the sensitivity of the device, the location of the signal, the nature of the object, and the basic personal data of the patient were noted (Graph 2).

After the HHMD examination, routine radiography of the entire gastrointestinal tract was conducted, as this is the gold-standard technique for detecting metal foreign objects. In most cases, only one radiographic image was captured while assessing the size, location, and any signs of serious complications pertaining to FB ingestion. We assessed the accessibility of foreign objects for upper endoscopic intervention using both HHMD and radiographic imaging, comparing and evaluating the accuracy of data gathered by the HHMDs. 

Statistical analysis was performed using IBM SPSS Statistics 24.0. Descriptive statistics and Chi-Square test were used for the analysis.

## 3. Results

**Figure 1:**
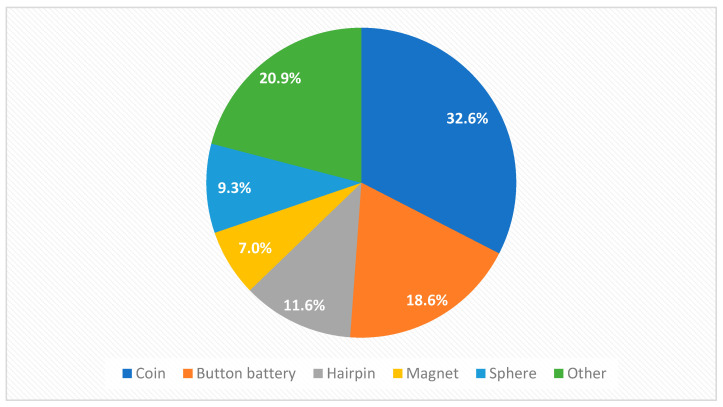
Types of ingested metal foreign bodies.

The data from 43 children (39.5% female; mean age: 4 years; min: 3 months; max: 11 years 4 months) that were referred to our emergency department were included in this study. Approximately two thirds (65%) of the children were referred on the day of suspected metal object ingestion, and almost all of them (92.5%) were referred within 7 days after suspected ingestion. The most common ingested objects were coins (32.6%), followed by button batteries (18.6%) and hairpins (11.6%) (Figure 1).

**Figure 2:**
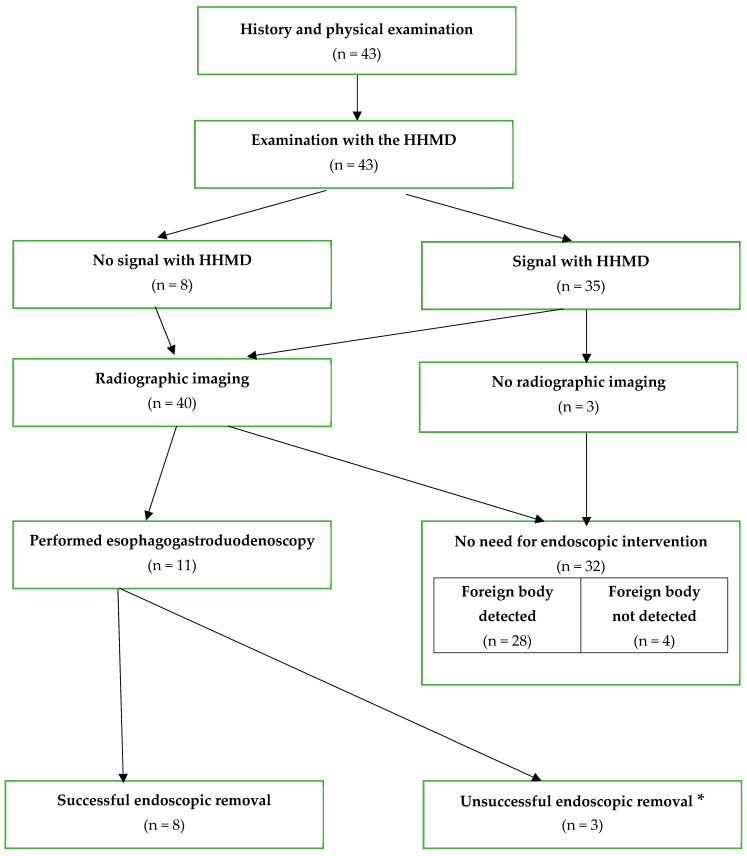
Flow chart of the management of patients suspected to have ingested a metal foreign object. * FB progressed beyond the reach of the endoscope.

All the children were first examined using the metal detector placed on the different parts of the body (neck, chest, abdomen) and in 35 children (81.4%) the signal was detected. Following this, radiographic imaging was conducted in 40 children. For the remaining three children (2 sphere ingestions and 1 coin), the metal detector indicated the location, which was not reachable for upper endoscopic intervention. Considering the non-harmful nature of the objects, radiographic imaging was not pursued, and no further intervention was planned (Figure 2). The examination with HHMD had a sensitivity of 89.7% and specificity of 100%.

**Figure 3:**
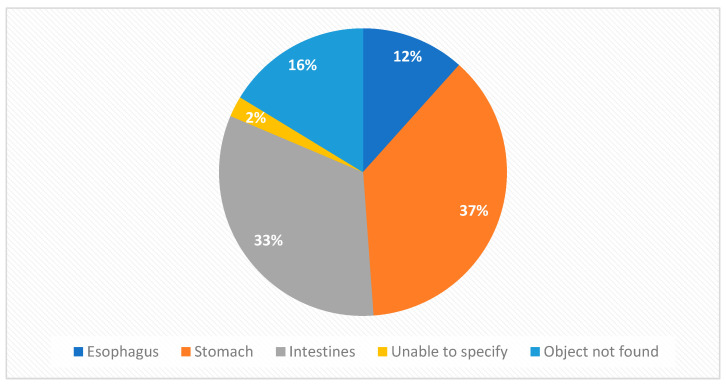
Locations of the foreign bodies according to radiographic imaging.

The most common location of the foreign object according to the radiographic imaging results was the stomach (37%), followed by the intestines (33%) and esophagus (12%) (Figure 3).

In the group of children who underwent both investigations (with the HHMD and via radiographic imaging; N = 40), the HHMD signal and radiographic imaging detected a foreign object in 32 cases (80%). In the other eight cases, no signal was noted on the HHMD; however, in four of these cases, radiographic imaging revealed a foreign object. Despite a clear history of FB ingestion as reported by the parents, in the remaining four cases, no FB was seen through radiography.

The signal from the metal detector correlated with the radiographic object location (reachable or unreachable) in 69.8% of cases (N = 30). According to the HHMD, 34.9% of foreign objects were accessible via upper endoscopic intervention, in contrast to the 51.2% identified through radiographic examination (*p* < 0.05). Successful endoscopic removal was performed for six of these patients (three coins, one sphere, and two hairpins; see Table 1). 

Endoscopic intervention was performed in 25.4% of cases (*N* = 11), predominantly for button batteries (*N* = 4) and sharp objects (hairpins, screw, and copper wires) located within endoscopic reach. In 72.7% of cases, the foreign body was removed, and in the remaining cases, the foreign object had already passed further in the gastrointestinal tract before the endoscopic intervention. 

Out of 43 children, 10 were referred to our department more than 1 day after presumed ingestion, and in three cases, neither the HHMD nor radiographic imaging detected a foreign body, so it was assumed that there was no foreign body ingested. In six cases, the foreign body was beyond endoscopic reach. In one case (3 days after ingestion), the foreign body (a coin) remained in the stomach and was endoscopically removed.

Assessing the efficiency of an HHMD for the detection of coins (*N* = 14), we observed a signal in 11 cases, and in all these cases, the presence of the coin was corroborated by radiographic imaging. In the remaining three cases where the HHMD did not register a signal, no foreign body was identified via radiographic imaging. Consequently, the sensitivity and specificity of the HHMD for coin detection were both 100%. Additionally, in three cases where an esophagogastroduodenoscopy was performed, the HHMD consistently indicated that the location of the FB was accessible via endoscopic examination.

In the group of other foreign bodies, the HHMD registered a signal in 24 out of 29 cases, with no signal in the remaining 5 cases. However, in the instances where the HHMD did not yield a signal, radiographic imaging showed the presence of an FB in four out of these five cases, as previously mentioned. Notably, one of these cases involved the subsequent endoscopic removal of a foreign body (copper wires). The sensitivity of the HHMD for detecting metal FBs other than coins was found to be 84.6%, with a corresponding specificity of 100%. 

The accuracy of the HHMD in detecting coins was better compared to that for the detection of other metal foreign bodies; however, no significant difference was observed.

## 4. Discussion

The ingestion of metal foreign bodies by children is a common concern, and several studies have investigated the utility of metal detectors in the detection of metal foreign bodies in children. The versatility of metal detectors allows for the detection of a broad spectrum of materials, encompassing both non-magnetic and magnetic substances such as iron, silver, lead, aluminum, copper, and brass. This adaptability positions metal detectors as valuable and efficient tools in clinical settings, offering a non-invasive alternative for identifying various metal foreign bodies [2,24]. However, the current ESPGHAN and NASPGHAN guidelines regarding the diagnostics and management of children after they have ingested a metal foreign body do not include any information on the use of HHMDs. Both recommend the use of radiographic studies, primarily those involving the classical radiographic imaging of the whole gastrointestinal track and, secondarily, CT imaging [1,25]. ESPGHAN Endoscopy guidelines state that there is not enough evidence to support the use of HHMDs in localizing ingested coins in children [19].

According to our study, the use of an HHMD appears to be a good non-invasive and non-irradiating examination for confirming the presence of a metal foreign object in children (with a sensitivity of 89.7% and a specificity of 100%). It is also useful for determining the absence of a foreign object in children that were referred to our department relatively late (more than 7 days after ingestion). 

Several studies have shown that coins constitute the most prevalent foreign bodies ingested by children, a trend affirmed in our study as well [4,5,26]. Aljasser et al. [4] demonstrated that an HHMD is a valuable surveillance tool post-confirmation of esophageal coin ingestion. Notably, strategic HHMD application resulted in a substantial 90.9% reduction in the need for repeat X-rays during monitored observation, effectively curbing radiation exposure and associated costs. Despite the acknowledged challenge of identifying coins in the mid-distal esophagus, the cited study reported a 100% success rate in coin detection using an HHMD [4]. The systematic review on coin detection using handheld metal detectors conducted by Lee et al. [27] demonstrated that the use of an HHMD is an accurate, radiation-free, and cost-effective method for identifying and localizing ingested coins in children. In a separate investigation by James et al. [2], a metal detector was successfully used in identifying swallowed metallic coins, exhibiting an accuracy level comparable to that achieved by plain radiographs. The same result was found in the study by Hamzah et al. [26], who reported that all coins were accurately identified by the handheld metal detector, aligning with the outcomes observed in our study. However, it is noteworthy that non-coin metal foreign bodies such as screws, needles, and stapler pins were not detected by the handheld metal detector [26]. Schalamon et al. [28] successfully identified all 32 coins ingested by children in their study using an HHMD. However, they encountered challenges, as 8 out of 15 non-coin metal foreign bodies, including two button batteries and a needle, went undetected. Similar difficulties have been noted by other investigators, reinforcing the message that HHMDs may not be consistently reliable in excluding the presence of metal foreign bodies other than coins [27,28,29,30]. In our study, there were four instances wherein the examination with an HHMD did not confirm the presence of a metal object (a battery, a magnet, a hairpin, or copper wires) in children, whose presence was later refuted through a radiographic examination. The wires were small, so they might not have produced a signal sufficient for the metal detector to detect. As for the other objects, we do not have a good explanation as to why they were not detected, apart from possible human error while performing the examination.

It is important to note that we routinely acquired one anterior-posterior radiograph and not two radiographic images in two projections. This was mainly due to a local decision made by the radiological unit to acquire only one projection radiograph in these cases. The ESGE/ESPGHAN and NASPGHAN guidelines recommend using routine two-projection radiographs for a better evaluation of the size and number of objects as well as a more precise evaluation of the location of the object. With these additional data, the decision to perform an endoscopic removal can be made more confidently [1,19].

In the study conducted by Nation et al. [23], the sensitivity of an HHMD for detecting metal foreign bodies was reported to be 89%, while its specificity was reported to be 100%. These findings closely align with the results of our study. However, in their research, positive HHMD indications had a 100% correlation with radiographic locations, constituting a notable difference from our study, where the correlation was 69.8%. Additionally, both studies identified four false-negative cases, emphasizing the necessity of cautious interpretation. Despite the high specificity obtained, the study by Nation et al. [23] highlights the importance of recognizing that HHMD sensitivity is not 100%. Consequently, a negative HHMD screening does not eliminate the need for using a standard radiograph to avoid missing metallic foreign bodies. An HHMD’s optimal performance is observed in the detection of coins, which constitute the most commonly ingested metal foreign bodies. However, its sensitivity may be compromised when dealing with smaller amounts of metal [23]. Our study further substantiates this point by revealing a lower sensitivity in detecting foreign bodies other than coins (84.6% compared to 100% for coins). 

In line with our own observations, the study conducted by Saz et al. [31] revealed that a handheld metal detector exhibited an 88.6% sensitivity and 100% specificity. Similar findings were noted in the research conducted by Sacchetti et al. [30], where a handheld metal detector demonstrated a sensitivity of 94% and a specificity of 100%. Additionally, the study of Guana et al. [32] maintained a high specificity of 95%, while the sensitivity displayed variability, resulting in an overall sensitivity of 63.2%. It is noteworthy that the cited study differentiated sensitivity rates for specific objects, reporting values of 79.5% for coins, 25.5% for batteries, and 56% for other objects [32].

These comparative studies emphasize the nuanced performance of HHMDs, emphasizing their strengths in certain contexts, particularly with respect to coins, and potential limitations in detecting various other metal objects. Understanding these variations is vital for the judicious integration of HHMDs in clinical practice to attain optimal diagnostic outcomes.

However, when it came to determining the location of the object and deciding if an endoscopic intervention is necessary, the data gathered with the HHMD were not satisfactory (with an accuracy rate of 69.8%). The final decision regarding the need for an endoscopic intervention should therefore be based on radiographic examinations.

We found some limitations of the use of HHMDs, especially compared with radiographic examinations. The precision and effectiveness of an examination using an HHMD can be impaired by a lack of sufficient knowledge of the apparatus and all its functions held by the physician performing the examination. Additionally, disregarding some of the prescribed examination details (e.g., not removing all metal objects, like parents’ jewelry, from a child’s surroundings or performing the examination on a bed with metal parts) can lead to faulty conclusions and results [2]. Lafferty et al. [33] also concluded that there are large variations in the techniques of HHMD use amongst physicians, who usually receive little formal training in their use. On the other hand, using HHMDs is technically not very demanding, and practically anyone (even non-medical personnel) can be taught how to properly handle this device and be informed of the limitations of this examination. It is worth considering using an HHMD in situations where radiographic examinations are not possible or cannot be performed in due time.

## 5. Conclusions

In conclusion, handheld metal detectors frequently exhibit strong correlations with radiography findings when detecting metal foreign objects. However, it is important to acknowledge their inherent limitations, particularly in confirming the precise location of an object. Handheld metal detectors fall short in providing comprehensive information that would allow physicians to make informed decisions regarding the necessity of an endoscopic intervention.

It is crucial to recognize that, despite their utility, handheld metal detectors do not offer sufficient granularity with which to guide clinical decisions with certainty. Therefore, while these devices serve as valuable screening tools, radiographic imaging retains its status as the gold standard for determining both the presence and precise location of ingested metal foreign objects in children. The comprehensive and detailed information provided by radiography remains indispensable in guiding clinical interventions and ensuring the optimal management of such cases.

## Figures and Tables

**Table 1:** Accuracy of HHMD in determining the accessibility of the ingested foreign object via endoscopic intervention.

						Number of Patients
HHMD confirms an endoscopically reachable foreign object	YES (*N* = 15)	Radiograph confirmed the presence of an endoscopically reachable foreign object	YES (*N* = 12)	Successful endoscopic intervention	Yes	6
					No	1
					Not performed	5
			NO (*N* = 3)	Successful endoscopic intervention	Yes	1
					No	0
					Not performed	2
	NO (*N* = 28)	Radiograph confirmed the presence of an endoscopically reachable foreign object	YES (*N* = 10)	Successful endoscopic intervention	Yes	2
					No	2
					Not performed	6
			NO (*N* = 18)	Successful endoscopic intervention	Yes	0
					No	0
					Not performed	18

## Data Availability

Data are unavailable due to privacy restrictions.

## References

[1] Kramer R.E., Lerner D.G., Lin T., Manfredi M., Shah M., Stephen T.C., Gibbons T.E., Pall H., Sahn B., McOmber M. (2015). Management of ingested foreign bodies in children: A clinical report of the NASPGHAN Endoscopy Committee. J. Pediatr. Gastroenterol. Nutr..

[2] James V., Hamzah H.B., Ganapathy S. (2018). Handheld Metal Detector Screening for Metallic Foreign Body Ingestion in Children. J. Vis. Exp. JoVE.

[3] Laya B.F., Restrepo R., Lee E.Y. (2017). Practical Imaging Evaluation of Foreign Bodies in Children: An Update. Radiol. Clin. N. Am..

[4] Aljasser A., Elmaraghy C.A., Jatana K.R. (2018). Utilization of a handheld metal detector protocol to reduce radiation exposure in pediatric patients with esophageal coins. Int. J. Pediatr. Otorhinolaryngol..

[5] Au A., Goldman R.D. (2021). Management of gastric metallic foreign bodies in children. Can. Fam. Physician Med. Fam. Can..

[6] Quitadamo P., Battagliere I., Del Bene M., Caruso F., Gragnaniello P., Dolce P., Caldore M., Bucci C. (2023). Sharp-Pointed Foreign Body Ingestion in Pediatric Age. J. Pediatr. Gastroenterol. Nutr..

[7] Chen Q.-J., Wang L.-Y., Chen Y., Xue J.-J., Zhang Y.-B., Zhang L.-F., Qian Y.-Z., Xiong Q.-X., Gao Z.-G. (2022). Management of foreign bodies ingestion in children. World J. Pediatr..

[8] Waltzman M.L. (2006). Management of esophageal coins. Curr. Opin. Pediatr..

[9] Lee J.H. (2018). Foreign Body Ingestion in Children. Clin. Endosc..

[10] Alfonzo M.J., Baum C.R. (2016). Magnetic Foreign Body Ingestions. Pediatr. Emerg. Care.

[11] Hong K.H., Kim Y.J., Kim J.H., Chun S.W., Kim H.M., Cho J.H. (2015). Risk factors for complications associated with upper gastrointestinal foreign bodies. World J. Gastroenterol..

[12] Pugmire B.S., Lim R., Avery L.L. (2015). Review of Ingested and Aspirated Foreign Bodies in Children and Their Clinical Significance for Radiologists. Radiographics.

[13] Demiroren K. (2023). Management of Gastrointestinal Foreign Bodies with Brief Review of the Guidelines. Pediatr. Gastroenterol. Hepatol. Nutr..

[14] Younger R.M., Darrow D.H. (2001). Handheld metal detector confirmation of radiopaque foreign bodies in the esophagus. Arch. Otolaryngol. Head Neck Surg..

[15] Seikel K., Primm P.A., Elizondo B.J., Remley K.L. (1999). Handheld metal detector localization of ingested metallic foreign bodies: Accurate in any hands?. Arch. Pediatr. Adolesc. Med..

[16] Ramlakhan S.L., Burke D.P., Gilchrist J. (2006). Things that go beep: Experience with an ED guideline for use of a handheld metal detector in the management of ingested non-hazardous metallic foreign bodies. Emerg. Med. J. EMJ.

[17] Salisu A.D. (2010). Metallic foreign body in esophagus: Are multiple radiographs necessary?. Ann. Afr. Med..

[18] Gooden E.A., Forte V., Papsin B. (2000). Use of a commercially available metal detector for the localization of metallic foreign body ingestion in children. J. Otolaryngol..

[19] Thomson M., Tringali A., Dumonceau J.M., Tavares M., Tabbers M.M., Furlano R., Spaander M., Hassan C., Tzvinikos C., Ijsselstijn H. (2017). Paediatric Gastrointestinal Endoscopy: European Society for Paediatric Gastroenterology Hepatology and Nutrition and European Society of Gastrointestinal Endoscopy Guidelines. J. Pediatr. Gastroenterol. Nutr..

[20] Kim J.K., Kim S.S., Kim J.I., Kim S.W., Yang Y.S., Cho S.H., Lee B.S., Han N.I., Han S.W., Chung I.S. (1999). Management of foreign bodies in the gastrointestinal tract: An analysis of 104 cases in children. Endoscopy.

[21] Chu K.M., Choi H.K., Tuen H.H., Law S.Y., Branicki F.J., Wong J. (1998). A prospective randomized trial comparing the use of the flexible gastroscope versus the bronchoscope in the management of foreign body ingestion. Gastrointest. Endosc..

[22] Nugud A.A., Tzivinikos C., Assa A., Borrelli O., Broekaert I., Martin-De-Carpi J., Saccomani M.D., Dolinsek J., Homan M., Mas E. (2023). Pediatric Magnet Ingestion, Diagnosis, Management, and Prevention: A European Society for Paediatric Gastroenterology Hepatology and Nutrition (ESPGHAN) Position Paper. J. Pediatr. Gastroenterol. Nutr..

[23] Nation J., Jiang W. (2016). The utility of a handheld metal detector in detection and localization of pediatric metallic foreign body ingestion. Int. J. Pediatr. Otorhinolaryngol..

[24] Arena L., Baker S.R., Baker L.A.A.S.R., Baines C.J., Miller A.B., Kopans D.B., Moskowitz M., Sanders D.E., Sickles E.A., To T. (1990). Use of a metal detector to identify ingested metallic foreign bodies. Am. J. Roentgenol..

[25] Mubarak A., Benninga M.A., Broekaert I., Dolinsek J., Homan M., Mas E., Miele E., Pienar C., Thapar N., Thomson M. (2021). Diagnosis, Management, and Prevention of Button Battery Ingestion in Childhood: A European Society for Paediatric Gastroenterology Hepatology and Nutrition Position Paper. J. Pediatr. Gastroenterol. Nutr..

[26] Hamzah H.B., James V., Manickam S., Ganapathy S. (2018). Handheld Metal Detector for Metallic Foreign Body Ingestion in Pediatric Emergency. Indian J. Pediatr..

[27] Lee J.B., Ahmad S., Gale C.P. (2005). Detection of coins ingested by children using a handheld metal detector: A systematic review. Emerg. Med. J..

[28] Schalamon J., Haxhija E.Q., Ainoedhofer H., Gössler A., Schleef J. (2004). The use of a hand-held metal detector for localisation of ingested metallic foreign bodies—A critical investigation. Eur. J. Pediatr..

[29] Muensterer O.J., Joppich I. (2004). Identification and topographic localization of metallic foreign bodies by metal detector. J. Pediatr. Surg..

[30] Sacchetti A., Carraccio C., Lichenstein R. (1994). Hand-held metal detector identification of ingested foreign bodies. Pediatr. Emerg. Care.

[31] Saz E.U., Arikan C., Ozgenc F., Duyu M., Ozananar Y. (2010). The utility of handheld metal detector in confirming metallic foreign body ingestion in the pediatric emergency department. Turk. J. Gastroenterol..

[32] Guanà R., Bianco E., Garofalo S., Castagno E., Cisarò F., Lemini R., Marchese V., Gennari F. (2023). Handheld metal detector versus conventional chest and abdominal plain radiography in children with suspected metallic foreign body ingestion: Can we safely abandon X-rays?. Minerva Pediatr..

[33] Lafferty M., Lyttle M.D., Mullen N., PERUKI (2021). Ingestion of metallic foreign bodies: A Paediatric Emergency Research in the United Kingdom and Ireland survey of current practice and hand-held metal detector use. J. Paediatr. Child Health.

